# Exploring the Relationship between Built Environment Attributes and Physical Activity in Lower-Income Aging Adults: Preliminary Insights from a Multi-Level Trial

**DOI:** 10.3390/ijerph21050607

**Published:** 2024-05-09

**Authors:** Arjan S. Walia, Abby C. King, Maria I. Campero, Dulce M. Garcia, Rebecca E. Lee, Astrid N. Zamora

**Affiliations:** 1 Stanford Prevention Research Center, Stanford University School of Medicine, Stanford, CA 94304, USA; awalia@stanford.edu (A.S.W.);; 2 Department of Epidemiology and Population Health, Stanford University School of Medicine, Stanford, CA 94304, USA; icampero@stanford.edu (M.I.C.);; 3 Center for Health Promotion and Disease Prevention, Edson College of Nursing and Health Innovation, Arizona State University, Tempe, AZ 85004, USA

**Keywords:** built environment, environmental justice, midlife adults, older adults, physical activity, walking

## Abstract

The built environment has been linked to physical activity (PA) behaviors, yet there is limited knowledge of this association among lower-income midlife and older adults who are insufficiently active. The present cross-sectional study utilized baseline data collected between October 2017 and November 2019 from a clustered randomized controlled trial to determine how built environment attributes were associated with PA behaviors among midlife and older adults (n = 255) residing in or near affordable housing sites (n = 10). At each site, perceptions of the built environment were collected and scored at the participant level via the abbreviated Neighborhood Environment Walkability Survey (NEWS-A), while objective built environment attributes were measured and scored by trained research staff using the Physical Activity Resource Assessment (PARA). Multiple PA behaviors—walking, total PA, and moderate-to-vigorous PA (MVPA) (min/wk)—were measured using the validated Community Healthy Activities Model Program for Seniors (CHAMPS) questionnaire. Adjusted linear regression models examined associations between NEWS-A measures and PA behaviors, and site-level correlations between PARA measures and PA behaviors were examined using Spearman’s rank correlations. At the participant level, adjusted models revealed that a one point increase in the NEWS-A aesthetics score was associated with a 57.37 min/wk increase in walking (β = 57.37 [95% CI: 20.84, 93.91], *p* = 0.002), with a similar association observed for street connectivity and MVPA (β = 24.31 min/wk [95% CI: 3.22, 45.41], *p* = 0.02). At the site level, MVPA was positively correlated with the quality of the features of local, PA-supportive environmental resources (ρ = 0.82, *p* = 0.004). Findings indicate that participant- and site-level measures of the built environment may play a role in promoting PA behavior among this demographic and similar populations. Results also suggest that improvements in aesthetic attributes and street connectivity, along with enhancing the quality of local, PA-supportive environmental resources, may be effective strategies for promoting physical activity among lower-income midlife and older adults.

## 1. Introduction

Engaging in physical activity (PA) plays a critical role in preventing and managing an array of chronic diseases ranging from cardiovascular disease (CVD) to frailty, diabetes, and dementia [1,2,3,4]. Staying active into midlife (ages 40 to 64 years) and older adulthood (ages ≥ 65 years) is also known to preserve perceived quality of life and cognitive function and improve resilience to unexpected adverse health events—in addition to reducing mortality risk [5,6,7,8,9]. In contrast, epidemiological studies have shown that sedentary behavior in these age groups is associated with an increased risk of falls, CVD, cancer, cognitive decline, and all-cause mortality [10,11,12]. Despite its importance in preventing disease throughout the life course, only 29.8% of older adults in the United States (US) are currently meeting national PA guidelines, which include walking—with that percentage further declining in recent years [13,14].

Growing evidence suggests that walking alone may provide health benefits for midlife and older adult populations [15,16]. For instance, results from case-control and cohort studies have shown that, for aging populations, regular walking can provide health benefits similar to leisure time moderate-to-vigorous physical activity (MVPA), and total PA (the sum of all activities regardless of intensity). Though moderate-to-vigorous forms of structured exercise and total PA are more often emphasized in interventions, walking as a form of PA is both accessible and relatively low-risk, especially for older adults [17,18]. Walking largely occurs for either transport—as a way of getting from one place to another (e.g., work, stores, school)—or recreation (e.g., walking a dog, hiking, walking for exercise). While recreational walking can also occur indoors (e.g., on a treadmill or indoor track), both types of walking typically take place outdoors and often within one’s immediate environment [15,16]. Given its ubiquity in daily life, walking is an important strategy to combat the growing levels of sedentary behavior, particularly for the aging population [17].

The built environment, encompassing the human-designed physical spaces with which people interact daily (e.g., sidewalks, parks, transportation systems), is a key determinant of PA behaviors and overall health and wellness [19,20,21]. Cross-sectional studies from North America, East Asia, and Europe have provided evidence of associations between objectively and subjectively assessed measures of the built environment with walking or other forms of PA [22,23,24,25,26,27]. However, although associations between the built environment and PA behavior have been well documented in the literature, it is important to acknowledge that many aspects of the built environment may be differentially linked to PA behaviors across different socioeconomic and age groups. For example, the safety of sidewalks and street crossings or the perceptions of car traffic and social conditions (e.g., cleanliness, crime) can have varying effects on people in different age groups [28].

Moreover, existing studies on the association between aspects of the built environment and PA have rarely focused on lower-income, insufficiently active aging (midlife-to-older) adult populations [29]. Given that midlife and older adults compose approximately half (48.5%) of the US population, knowledge of the connections between the physical surroundings and PA behaviors in this population is critical to creating effective interventions and policy-level changes that can encourage PA and, therefore, healthy aging [30,31].

To better understand the PA behaviors of this understudied population of lower-income midlife and older adults, we conducted a cross-sectional analysis examining how subjective perceptions and objective measures of the built environment were associated with minutes per week of walking, total PA, and moderate-to-vigorous PA behaviors among a sample of community-dwelling, insufficiently active adults living in or near ten affordable public housing sites in the San Francisco Bay Area. In using both individual- and site-level outcomes, we draw from the socio-ecological model, which maintains that not only individual factors but also social, environmental, and other upstream determinants are important to understanding and promoting health [32,33]. Thus, our study focused on both individual- and community-level factors to mirror the interactions between multiple levels as theorized by the socio-ecological model. The secondary aim of this analysis was to examine differences between the midlife and older adult subgroups in this cohort, given the known differences in PA behavior across life stages [34,35]. In addition, a focal reason for wanting to focus on this demographic of older adults residing in or near affordable housing sites (an indicator that they were likely on fixed incomes) within Santa Clara and San Mateo counties within the San Francisco Bay Area related to participants living in two of the wealthiest counties within in the US [36], which presents a unique context for our study. Although wealth is strongly associated with access to resources, better health outcomes, and healthy aging [37], it is often assumed that wealth is distributed or accessible to all residents within a particular locale. However, in areas with high inequality such as the Bay Area, this is often not true, and the inequitable environmental conditions faced by aging adults residing in affordable dwellings may be overlooked with this assumption. Our focus on this demographic underscored the importance of investigating the impacts of the local built environment in such communities, where residents are potentially subject to neglect despite living in affluent locales.

Based on the existing literature on the general adult population and older adults that demonstrates the links between neighborhood walkability and PA, we hypothesized that participant-level perceptions of walkability would be positively associated with PA behaviors. Furthermore, we hypothesized that site-level measures of the quality of PA-relevant resources would be associated with PA. By utilizing both individual and community measures within the framework of the socio-ecological model, we provide a more comprehensive view of how the built environment is associated with PA behaviors within this demographic of lower-income, insufficiently active aging adults.

## 2. Materials and Methods

### 2.1. Study Design

Study participants were enrolled in the National Institutes of Health (NIH)-funded Steps for Change (SFC) trial, a 24-month single-blind, cluster-randomized controlled parallel trial comparing the effectiveness of an evidence-based participant-level PA intervention with or without a built environment-focused intervention to facilitate neighborhood walkability. The primary focus of the intervention was to increase the number of minutes per week of walking, an easily accessible and popular form of PA, particularly for insufficiently active adults [38]. Complete study details have been published previously [39]. The collection of the baseline data occurred between October 2017 and November 2019. The trial was registered at Clinicaltrials.gov (#NCT03041415) and was approved by the Stanford University Institutional Review Board.

In brief, the study recruited eligible, low-income participants living in or around ten senior affordable public housing sites in Santa Clara and San Mateo counties of Northern California, recognized as two of the wealthiest counties in the US (See Table 1 for a list of housing sites and the number of participants enrolled at each site) [40,41]. Our selection of the San Francisco Bay Area as the study location was guided by practical considerations. Given the strong pre-existing ties with local community organizations, we already had a robust research infrastructure in this region and could fully leverage these partnerships for the mutual benefit of communities and researchers—in line with the principles of community-engaged participatory research (CBPR) [42]. By leveraging these deep-rooted community links and the expertise of our partners, we could ensure an adequate pool of eligible study participants that was representative of the racial/ethnic diversity and geographic divisions of the Bay Area. 

The participants included in the study comprised lower-income, insufficiently active midlife and older adults who lived in or near these affordable housing sites. The designation as “low income” is derived from the US Department of Housing and Urban Development income limits, which bases its cutoff on the median incomes of a given county and number of people in the household [43]. Individuals living in or around these housing sites were eligible to participate if they were: (a) age 40 or older; (b) able and willing to increase their walking time in their neighborhood; (c) able to safely engage in moderate forms of PA such as walking, as indicated by the Physical Activity Readiness Questionnaire (PAR-Q); (d) able to read and understand English or Spanish sufficiently well to consent and participate in all study procedures; and (e) planning to live in the area for the subsequent 24 months [44]. The full exclusion criteria can be found in the SFC methods publication [39].

#### 2.1.1. Built Environment Measures

Drawing from the socio-ecological model for health promotion, this investigation employed a multi-tiered approach to assess various constituents of the participants’ built environment. This model proposes that social, environmental, and individual factors influence one another and synergistically affect individual health behaviors and population health [32]. Focusing on measures at the individual (participant) and community (housing site) levels enabled a more comprehensive understanding of the characteristics of the built environment (See Table 2 for information about the instruments utilized).

At the participant level, local built environment perceptions were measured using the Neighborhood Environment Walkability Survey—abbreviated form (NEWS-A), which uses a socio-ecological approach to measure individual perceptions of the built environment [45]. The abbreviated version, composed of 54 questions across 12 domains, was developed from the full-length NEWS questionnaire and cross-validated with the original [46]. This instrument measures individual perceptions of the local built environment factors that have been shown to affect PA in each of the 12 domains: types of residences, stores/facilities/other things in the neighborhood, access to services, street connectivity, places for walking/cycling, aesthetics, traffic hazards, crime, lack of parking, cul-de-sacs, hilliness, and barriers to walking. NEWS-A was collected at baseline, and the overall continuous summary scores for each domain were calculated using the relevant Likert scale questions according to previously reported methods [46].

At the community (site) level, the Physical Activity Resource Assessment (PARA), a systematic, researcher-initiated checklist instrument with high inter-rater reliability, was used to catalog and rate the quality of the local facilities and resources that influence PA in a neighborhood [47]. This instrument surveyed each of the three following domains with 37 total items to determine the quality of local physical activity resources (PAR): features for PA (13 items, e.g., trails for walking or biking, pools, exercise stations), visitor amenities (12 items, e.g., drinking fountains, landscaping efforts, lighting), and incivilities (12 items, e.g., auditory annoyance, lack of or overgrown grass, vandalism). Features and amenities were rated as not applicable (0), poor (1), mediocre (2), or good (3), whereas the prevalence of incivilities was rated as not applicable (0), little (1), some (2), or a lot (3) [47]. Research staff first identified possible PARs, including parks, trails, community centers, and sports facilities within a 1-mile radius of each SFC housing site using searches in Google Maps. Then, trained study staff visited these sites and completed the PARA checklist for each site. Previous studies have shown high inter-rater reliability (k values > 0.77) [47]. In the SFC trial, a total of 293 PARs were recorded across the ten sites surveyed (See Table S2 for descriptive statistics for the PARs near each housing site).

**Table 2:** Instruments utilized to measure physical activity and the built environment.

Tools and Measures	Summary	Domains	Citation
Physical Activity Resource Assessment (PARA)	A researcher-initiated checklist instrument consisting of 37 questions that characterize and rate the quality of PA resources in local neighborhoods.	Community Environment	Lee et al., 2005 [47]
Neighborhood Environment Walkability Survey—Abbreviated Form (NEWS-A)	A participant-completed survey consisting of 54 questions across 12 domains that measure individual perceptions of the local physical and social environment.	Community Environment	Cerin et al., 2006 [46]
Community Health Activities Model Program for Seniors (CHAMPS)	An interviewer-administered survey which asks participants to report how frequently and for what duration they usually participated in 41 activities of various intensities over the past four weeks.	Physical Activity	Stewart et al., 2001 [48]

Abbreviations: PA = physical activity.

#### 2.1.2. Physical Activity Behavior Measures 

Physical activity was measured using the interviewer-administered, self-reported Community Healthy Activities Model Program for Seniors (CHAMPS) questionnaire, originally developed and validated for adults aged 50 and older, but has been shown to be valid for estimating the PA behaviors of other adult populations, including those younger than 50 [48,49,50]. The CHAMPS questionnaire was administered by trained personnel in English or Spanish according to the participant’s preference. The questionnaire asked participants to report whether they engaged in 41 different activities of various intensities in the survey in a typical week over the past four weeks. If they had participated in that activity, they were asked how many times per week and the approximate minutes per week that they engaged in that activity in a typical week. By summing different questions in the CHAMPS questionnaire, we calculated the typical weekly totals for the three PA behaviors of interest: total walking, total PA, and total moderate-to-vigorous PA (MVPA), all measured in minutes per week (min/week).

#### 2.1.3. Covariates

At baseline, participants self-reported sociodemographic information, including age, sex, education level, income, and race and ethnicity [51]. Those who self-identified as being of Latino or Hispanic ethnicity were grouped into a single “Latino” category regardless of self-identified race. This grouping was utilized to reflect the understanding that many Latino/a people do not believe that traditional racial categories adequately represent their lived experiences [52,53]. The racial categories used in analyses—White, Asian, Black, and Native—represent the non-Hispanic people who self-identified as part of those respective racial groups. Participants who reported two or more races were considered a separate group, which was classified as multiple races. In the descriptive statistics in Table 1, however, they were also counted in each racial group with which they self-identified [54]. In-person height and weight measurements were taken by trained research staff twice during the assessment visit using standard clinical assessment protocols and then averaged to calculate body mass index (BMI) [49].

### 2.2. Statistical Analyses

#### 2.2.1. NEWS-A Analysis

**Figure 1:**
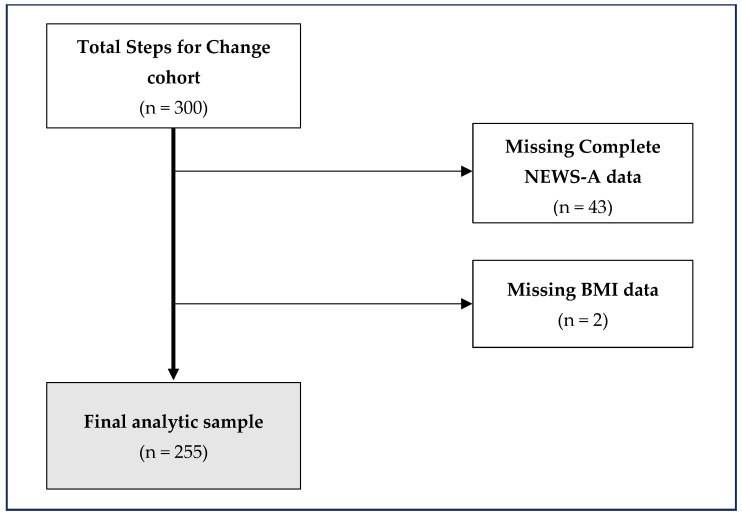
Study flow of the present secondary analysis for NEWS-A from the Steps for Change cohort.

Analysis of the perceptions of the built environment as measured by the NEWS-A questionnaire was performed on a subset of participants enrolled in the SFC trial. At baseline, individuals with missing data for NEWS-A (n = 43) or BMI (n = 2) were excluded from the current analysis (See Figure 1 for study flow chart). Table 1 summarizes the demographic and clinical characteristics of the full SFC cohort (n = 300) as well as the analytic sample (n = 255). Statistical differences between the analytic sample and the full SFC cohort were assessed using chi-square tests and two-sample *t*-tests. Table S1 presents crude associations between demographic variables and the three PA outcomes, utilizing Analysis of Variance (ANOVA) to assess differences. 

At the participant level, we used linear regression to examine the associations of participants’ perceptions of the built environment with each PA measure: walking, total PA, and MVPA. Building unadjusted and adjusted models for the three PA outcomes resulted in six distinct models. To build these, separate linear regression models with each of the 12 NEWS-A domains were created for each PA outcome. These models also included a variable for housing site to account for clustering and, for the adjusted models, the sociodemographic covariates used in the final models (age, sex, highest educational attainment, race/ethnicity, and BMI). To demonstrate, when constructing the unadjusted (1) and adjusted (2) regression models, the univariate models were as follows:

Y = β_o_ + β_X_ + β_site_ (1)

Y = β_o_ + β_X_ + β_site_ + Sociodemographic covariates (2)

where each NEWS-A domain was separately included for X, and each PA outcome for Y. NEWS-A domains that met *p* ≤ 0.10 in these individual models were included in the combined model for the respective PA outcomes.

Pearson’s correlation tests were conducted between each of the 12 NEWS-A domains to determine the extent of collinearity between the terms in these models. If two variables had moderately strong or greater correlation (|ρ| > 0.40), the less significant variable (i.e., had a higher *p*-value) in the preliminary combined model was removed. The variables that remained constituted the final regression model.

In addition to unadjusted models that only included the variable for housing site to account for clustering, we ran adjusted regression models. Adjusted models were selected a priori and informed by previous literature on the factors associated with PA and the built environment in adults, which included age, sex, education level, race/ethnicity, and BMI [55,56,57,58].

We report β coefficients and 95% confidence intervals (CI) representing the change in the PA outcome (min/wk), corresponding to a one point increase in the respective NEWS-A domain score. Based on *a priori* knowledge of differences in PA behaviors and mobility needs between people in these two life stages, we also conducted age group stratified analyses and examined whether there were differences in associations within the midlife (40–64 years old; n = 78) and older adult (≥65 years old; n = 177) subgroups [22,28]. Although we did not find a significant interaction between age group and each NEWS-A domain, we nonetheless ran stratified analyses for descriptive purposes.

Given that the present first-generation investigation was considered hypothesis-generating in nature, results were considered significant at *p* < 0.05. All analyses were performed in SAS (online version, Cary, NC, USA).

#### 2.2.2. PARA Analysis

Objective built environment variables gathered through the PARA neighborhood audit were analyzed by taking average scores from the PARA instrument and PA outcomes for each housing site. The physical activity resources (PARs) were categorized depending on their function and the resources available, and seven categories describing the main purpose of that PAR were created by combining similar ones from the PARA instrument: park/trail/greenspace, sports facility/fitness club, community center, church, school, plaza, or a combination of these PAR types (See Table S2 for descriptive statistics). To obtain summary quality scores for the features, amenities, and incivilities domains, we first calculated averages for each PAR by summing the scores from each question in that domain and dividing by the number of questions (13 for features and amenities, and 12 for incivilities). Then, these scores were averaged across all the PARs surrounding a housing site to get an overall mean for that PARA domain around each site. It is important to note that scores of 0 (indicating absence) were included in these averages, resulting in a possible range of 0 to 3. Since the unit of measurement for this community-level analysis was the housing site, we calculated the mean total walking, mean total PA, and mean MVPA minutes per week for each housing site (n = 10) by averaging the respective PA outcomes for all participants within that housing site (See Table S2 for means of these outcomes at each site).

Next, we used Spearman’s rank correlations (*ρ*) between the normalized quality scores for each PARA domain and mean PA outcomes to examine correlations at the housing site level. Spearman’s nonparametric correlation coefficients were utilized due to the small size of this sample (n = 10 housing sites) and the lack of a normal distribution in the averages of the PA outcomes. Both the PARA quality scores and PA means were operationalized as continuous measures.

## 3. Results

### 3.1. NEWS-A Participant-Level Findings

Among the analytic sample, the mean (SD) age was 69.4 (10.5) years and 74% of the sample identified as female. Sixty-one percent of the sample identified as non-Hispanic white, followed by about a fifth (19%) identifying as Latino/Hispanic. Annual income showed a wide distribution, with about 46% (118) making less than $75,000 annually. There were no statistically significant differences across baseline participant characteristics between the full SFC sample (n = 300) and the analytic sample (n = 255) [Table 1].

At the participant level, results from unadjusted and adjusted linear regression models between NEWS-A domains and PA outcomes revealed multiple statistically significant associations (Table 3). Among the analytic sample, results revealed that the perceived aesthetics summary score was positively associated with walking (β = 57.37 min/wk [95% CI: 20.84, 93.91], *p* = 0.002). After stratifying by age group, results remained statistically significant for both older adults (β = 51.00 min/wk [95% CI: 2.20, 99.79], *p* = 0.04) and midlife adults (β = 85.79 min/wk [95% CI: 14.81, 156.77], *p* = 0.02), indicating no differences in the association between the two age groups. In the adjusted model for MVPA, a one point increase in the summary score for street connectivity was significantly associated with a 24 min/week increase in MVPA (β = 24.31 min/wk [95% CI: 3.22, 45.41], *p* = 0.02). After stratifying by age group, the positive association persisted among older adults (β = 34.09 min/wk [95% CI: 8.91, 59.28], *p* = 0.008), while a non-significant negative association emerged in the midlife adult group (β = −19.09 [95% CI: −63.54, 25.35], *p* = 0.39).

### 3.2. PARA Site-Level Findings

Supplemental Table S2 summarizes the descriptive statistics for all 293 PARs across the ten housing sites collected via the PARA observational audit instrument. Briefly, results revealed that site H had the largest number of PARs (n = 45) but also the highest score for incivilities (0.46 out of a maximum of 3). Site I had the highest scores for the quality of features (0.71) and amenities 1.43) relative to the other housing sites.

Table 4 presents the correlations between each PA outcome and individual PARA domain scores. The results revealed that MVPA was positively correlated with the PARA features quality score (ρ = 0.82, *p* = 0.004), indicating that more and higher quality PA-friendly features were associated with higher levels of MVPA. Smaller, non-significant associations were observed for the amenities quality and incivilities severity domains and the three PA variables.

## 4. Discussion

In the present exploratory cross-sectional study, we found that both the participant-level self-reported measures and site-level researcher-assessed measures of the built environment were correlated with PA behaviors within a generally lower-income population of insufficiently active midlife and older adults. Participant-level perceptions of neighborhood aesthetics and street connectivity were associated with walking and MVPA behaviors, respectively. At the same time, site/community-level data collected by researchers revealed that MVPA was positively associated with the quality of the PA features found in neighborhood PARs. However, the inconsistency in associations found between the built environment and physical activity behaviors when employing objective measures signifies that these relations may be population and context-specific. These findings thus illuminate the unique challenges faced by lower-income midlife and older adults, emphasizing the importance of embedding environmental justice (EJ) frameworks that shed light on inequitable distribution and access to environmental spaces that are tied to PA behavior. By acknowledging variations in individual and community-level perceptions and their influence on physical activity outcomes, we can construct more effective, context-specific interventions that highlight the principles of environmental justice. This approach would fully recognize the heterogeneity within such demographics and allow for more equitable physical activity-promoting interventions and aging-friendly policies.

In general, our participant-level findings on the importance of aesthetics and street connectivity align with the existing literature about the influence of the built environment on PA behaviors in aging adults. Individual perceptions of the built environment, as measured primarily by NEWS-A or its full-length precursor, NEWS, have consistently shown associations with PA outcomes [59]. For example, a recent meta-analysis by Stearns et al. (2023) reported significant positive associations between total PA and total walking with greenery and aesthetically pleasing scenery, as well as a positive association between street connectivity and walking for transportation [22]. Both quantitative and qualitative studies have also indicated the importance of aesthetic components such as shady trees and the presence of attractive features in facilitating PA behaviors, especially for older adults [28,60]. In a study that included 17 cities around the world, with mean ages ranging from 34.0 to 46.6 years, Kerr et al. (2016) found that adults had significantly greater odds of achieving the recommended 150 min/week or more of walking for transport with greater perceived aesthetics and street connectivity, along with other variables from NEWS [61].

While there were many similarities between this lower-income, insufficiently active aging population and those that have been studied previously, several domains of NEWS-A—such as traffic, crime, and hazards that other studies have found to be important—were not included in the final regression models in this investigation due to lack of significance [22,62,63,64]. Comparing the findings of our study with those of other researchers elucidates that perceptions of aesthetics and street connectivity seem to be similarly important to PA in this specific insufficiently active midlife and older adult population as well as in the general aging population [62,63,65]. In contrast, certain other perceptions of the physical and social environment that have been noted as important in other studies of older adults were found to be less important in our lower-income sample of midlife and older adults. These include safety [66] and pedestrian infrastructure [67]. Although it is unclear why these differences emerged, the authors posit that this contrast may stem from a variety of socioeconomic and/or contextual factors specific to the population under study, highlighting the broad diversity within midlife and older adults that can influence their priorities and perceptions. For example, although our study did not explore the detailed reasons behind these varied perceptions, we hypothesize that other factors may have overshadowed concerns, such as safety or pedestrian infrastructure, among the study sample. In addition, habitual exposure to certain living conditions or experiences, such as elevated crime rates or the absence of pedestrian-friendly amenities, may have led to a normalization of these issues, thus reducing their perceived significance. These posited interpretations not only suggest possible explanations but also emphasize the necessity for further research that specifically addresses these disparities. Moreover, these specific similarities and differences provide an important backdrop for future research and policy interventions and highlight the importance of local contexts in more clearly understanding the circumstances at work in a particular locale.

In line with our finding about the importance of the quality of features for MVPA, Stearns et al. found in their meta-analysis that recreational facilities and parks/open spaces—which would fall under the features category of the PARA—were positively associated with PA [22]. More broadly, research at the community level utilizing objective measures—whether PARA or other neighborhood-level audits such as geographic information system (GIS) mapping—has found inconsistent associations between the built environment and PA behaviors, especially walking [24,25,68,69,70,71]. This suggests, as indicated in the current investigation that these relations may be specific to different populations living in varying environmental conditions.

Taken together, our findings of the importance of aesthetics and street connectivity, as well as quality of PARs’ features, revealed that insufficiently active, lower-income midlife and older adults may have somewhat distinct responses to the built environment relative to the more general midlife and older adult populations that are often the targets of investigation. These results, along with the existing literature, shed light on the impact of urban planning and public health policy and underscore the importance of aesthetics, including green spaces and connected streetscapes, in developing more walkable, health-promoting, and aging-friendly communities.

### 4.1. Promoting Environmental Justice in Future Research and Policy

In this study, we focused on a significant yet often overlooked segment of society: lower-income, older adults residing within two of the wealthiest counties in the US. Our key findings, which align with prior research among the general adult population, highlighted the influence of both personal perceptions and observable neighborhood characteristics on physical activity behaviors within this unique demographic. Specifically, we found that participants’ perceptions of neighborhood aesthetics and street connectivity were notably linked to walking and moderate to vigorous physical activity (MVPA), respectively. Moreover, our site/community-level data revealed a positive association between MVPA and the quality of physical activity features within neighborhood parks and recreation areas. Employing environmental justice (EJ) frameworks could be optimal for translating these insights into physical activity-promoting interventions and aging-friendly policies. The EJ approach emphasizes the equal right to environmental protection and provision regardless of socioeconomic status, and our results underscore the importance of such fairness in addressing neighborhood aesthetics, street connectivity, and park quality to promote physical activity and improve health outcomes [72,73,74]. Importantly, this approach is crucial for lower-income populations, such as our study participants, who are often overlooked despite residing within affluent locales. By emphasizing these pivotal findings in the context of the EJ framework, we believe our study contributes essential insights to the application of socio-ecological and environmental justice principles in public health research and intervention design. Future research interventions that center EJ would ensure greater access to health-enhancing environments, thereby mitigating health disparities. Frameworks for community-engaged research, such as CBPR, can also contribute to EJ’s emphasis on meaningfully involving local communities in these changes, ensuring that such interventions are based on the community’s experiences and, therefore, address their specific needs [42,75,76,77]. Given that a previous study found that lower-income women differentially benefitted from greater availability of high-quality PARs nearby, such socio-ecological changes in line with EJ principles may prove especially beneficial to addressing health inequities and environmental injustices in lower-income communities such as those in the present study [78].

Efforts to improve both the aesthetics and street connectivity of neighborhoods can draw from the principles of EJ to create more walkable communities, thus promoting PA. Our finding that the perception of neighborhood aesthetics was significantly associated with walking in this population suggests that cost-effective improvements to the aesthetic components of the built environment (e.g., planting more street-level trees and greenery, replacing unused areas with parks, or beautifying existing green spaces to better fit the needs of communities) could be effective changes at the policy level to promote PA [79,80,81,82]. Though limited research currently exists to demonstrate that ecological changes to a neighborhood’s aesthetics can improve PA outcomes, evidence of the benefits of “pop-up parks”—temporary parks created by bringing exercise equipment and potted greenery into greenspace-deficient areas—on PA behaviors suggests the potential promise of such built environment policy interventions [83,84]. Moreover, designing “age-friendly cities” that support and enable people to age actively through built and social environment improvements, as the World Health Organization has proposed, is one possible framework to ensure that the needs of the growing population of older adults globally are not left behind in campaigns to improve the walkability of urban environments [20,85,86]. Based on our findings linking street connectivity to MVPA behavior in this insufficiently active, aging population, prioritizing the safety of street crossings and intersections (e.g., longer crossing times at intersections, crossing countdowns with both audio and visual cues, neckdown or bulb-out crosswalks) and providing more and safer possible routes for people to take when engaging in PA in their neighborhoods may be good starting points in such policy work.

### 4.2. Strengths and Limitations

The present study adds to our understanding of how built environment factors relate to PA behaviors in insufficiently active midlife and older adults and draws attention to both individual- and community-level influences. A key strength was that our research methodology, which combined subjective and objective measures of the built environment, offers a more comprehensive view that highlights the experiences of aging adults in their local environments—an approach that has not commonly been explored in existing studies. In line with the socio-ecological model, this methodological innovation underscores the importance of considering both the personal perceptions of individuals and objective measures of the built environment, which together can significantly influence PA behaviors.

As there are several notable limitations to our investigation, we advise caution when interpreting the results. Most importantly, the cross-sectional design and small sample size of the site-level analysis using the PARA (n = 10 sites) limits the power and predictive utility of such an analysis, making correlations exploratory at best and lacking in temporality. Although we examined how individual perceptions and objective measures of the built environment are related to physical activity, we could not perform a multi-level modeling analysis. This approach could potentially offer a more nuanced understanding of how these variables interact concurrently to influence physical activity behaviors. Our decision to analyze individual perceptions separately was mainly due to statistical considerations, particularly the relatively small sample size for the objective measures. We acknowledge that a multi-level model, integrating both objective measures and perceptions, would be a valuable extension of this work. However, incorporating such a framework necessitates considerably larger sample sizes to maintain adequate statistical power and validity. The cross-sectional nature of our study design thus does not permit the drawing of causal inferences. Moreover, our method of scoring the PARA quality, by averaging all the questions in each domain regardless of the absence of a particular component (score of 0), may not adequately represent the overall quality of the PARs surrounding a housing site compared to methods only considering present components (i.e., a score of 1 or greater). For example, if a PAR has a “good” soccer field with a score of 3 but lacks other features, then the overall features quality score may appear deceivingly low despite the appeal of such a soccer field. Importantly, the presence of physical activity resources (PARs) themselves does not necessarily mean that community members are utilizing those resources. Future investigations using the PARA instrument may be enhanced by complementing it with use and access data that can shed light on how often such resources are being utilized [87]. For the participant-level analysis, the small sample size of midlife adults (n = 78) likely resulted in relatively low power to detect associations in this subgroup. Finally, the overrepresentation of some demographic subgroups (e.g., females) compared to the general population may limit the generalizability of these findings.

## 5. Conclusions

In summary, the present study aimed to better understand the associations between physical activity behaviors and subjective and objective built environment measures in a population of lower-income, insufficiently active midlife and older adults from the San Francisco Bay Area. Our findings successfully highlighted the positive links between high-quality PA resources and favorable perceptions of neighborhood aesthetics and street connectivity with physical activity. Our findings underscore the continued need for further research that embraces a socio-ecological approach by including both personal perceptions and objective features of the built environment as important factors linked with PA behaviors. By fostering environments that encourage active living and healthy aging in line with the principles of environmental justice, future interventions can be tailored to enhance neighborhood conditions, prioritize key environmental improvements, and potentially reduce health disparities.

## Figures and Tables

**Table 1:** Demographic and clinical characteristics of overall SFC sample versus analytic sample.

	Mean (SD)	
	Overall SFC Sample (N = 300)	Analytic Sample (N = 255)
**Sex, n (%)**		
Male	79 (26)	67 (26)
Female	221 (74)	188 (74)
*p* = 0.99		
**Race/ethnicity, n (%)**		
African American/Black, non-Hispanic	10 (3)	9 (4)
Asian, non-Hispanic	53 (18)	42 (16)
Native American, non-Hispanic	2 (1)	2 (1)
White, non-Hispanic	183 (61)	155 (61)
Refused race, non-Hispanic	1 (<1)	1 (<1)
Latino/Hispanic ethnicity	55 (18)	49 (19)
Two or more/multiple races	4 (1)	3 (1)
*p* = 0.99		
**Education, n (%)**		
Less than high school	7 (2)	6 (2)
High school or equivalent	52 (17)	43 (17)
College	157 (52)	131 (51)
Post-graduate	83 (28)	74 (29)
Other	1 (<1)	1 (<1)
*p* = 1.0		
**Annual income, n (%)**		
<$5000	2 (1)	2 (1)
$5000–9999	9 (3)	5 (2)
$10,000–14,999	21 (7)	17 (7)
$15,000–24,999	31 (10)	27 (11)
$25,000–34,999	18 (6)	14 (5)
$35,000–49,999	27 (9)	24 (9)
$50,000–74,999	35 (12)	29 (11)
>$75,000	85 (28)	79 (31)
Do not know or refused	72 (24)	58 (23)
*p* = 0.99		
**Age, mean (SD)**	69.5 (10.3)	69.4 (10.5)
*p* = 0.91		
**BMI category, n (%)**		
Normal (19–24)	60 (20)	48 (19)
Overweight (25–29)	102 (34)	87 (34)
Obese (30–39)	118 (39.3)	104 (40)
Extreme Obesity (40–54)	18 (6)	16 (6)
*p* = 0.98		
**Housing site, n (%)**		
Site A	22 (7.3)	14 (5)
Site B	21 (7)	15 (6)
Site C	31 (10)	21 (8)
Site D	14 (5)	14 (5)
Site E	16 (5)	16 (6)
Site F	17 (6)	15 (6)
Site G	41 (14)	35 (14)
Site H	42 (14)	32 (13)
Site I	47 (16)	47 (18)
Site J	49 (16)	46 (18)
*p* = 0.96		

Abbreviations: SD: standard deviation; N = sample size; SFC = Steps for Change; *p* values from chi-square test or *t*-test.

**Table 3:** Estimates and confidence intervals (CIs) from linear regression analysis of three physical activity (PA) outcomes stratified by age group.

	Total Sample (n = 255)		Older Adults [≥65 yrs] (n = 177)		Midlife Adults [40–64 yrs] (n = 78)	
	Unadjusted ^#^ β (95% CI)	Adjusted ^†^ β (95% CI)	Unadjusted ^#^ β (95% CI)	Adjusted ^†^ β (95% CI)	Unadjusted ^#^ β (95% CI)	Adjusted ^†^ β (95% CI)
Total walking (min/wk)						
Residential density	---	0.25 [−0.08, 0.58]	---	0.27 [−0.12, 0.67]	---	−0.10 [−0.92, 0.71]
Stores, facilities, and other things in the neighborhood	−9.59 [−35.40, 16.22]	---	−32.05 [−65.24, 1.14]	---	41.35 [−0.05, 82.75]	---
Places for walking/cycling	30.16 [−7.53, 67.86]	30.47 [−7.13, 68.07]	19.95 [−28.79, 68.68]	30.22 [−19.20, 79.65]	49.00 [−8.79, 106.79]	33.85 [−31.29, 99.00]
Aesthetics	55.71 ** [20.24, 91.18]	57.37 ** [20.84, 93.91]	48.11 ** [2.30, 93.93]	51.00 ** [2.20, 99.79]	79.77 ** [22.83, 136.71]	85.79 ** [14.81, 156.77]
Total PA (min/wk)						
Access to services	40.94 [−14.87, 96.75]	37.49 [−18.78, 93.76]	44.74 [−22.82, 112.30]	36.17 [−32.57, 104.92]	58.07 [−47.01, 163.16]	46.68 [−69.02, 162.38]
Aesthetics	65.76 [−8.71, 140.24]	51.60 [−24.36, 127.56]	71.35 [−21.56, 164.26]	52.36 [−43.38, 148.10]	101.61 [−31.48, 234.70]	125.45 [−35.69, 286.58]
Total MVPA (min/wk)						
Lack of parking	−12.60 [−28.13, 2.93]	---	−12.58 [−32.17, 7.00]	---	−11.11 [−38.84, 16.62]	---
Street connectivity	21.78 ** [1.22, 42.34]	24.31 ** [3.22, 45.41]	32.14 ** [7.49, 56.78]	34.09 ** [8.91, 59.28]	−17.23 [−58.75, 24.29]	−19.09 [−63.54, 25.35]
Aesthetics	16.48 [−7.45, 40.41]	14.76 [−9.77, 39.29]	14.42 [−15.48, 44.32]	8.70 [−22.54, 39.93]	33.58 [−9.94, 77.09]	43.71 [−8.54, 95.96]

Abbreviations: MVPA = moderate-to-vigorous physical activity. ^#^ Includes housing site to account for clustering. ^†^ Adjusted for age, sex, education level, race/ethnicity, BMI, and housing site. ** *p* < 0.05.

**Table 4:** Spearman’s rank correlation coefficients between baseline physical activity (PA) variables and PARA domains (N = 10).

	Walking (ρ)	Total PA (ρ)	MVPA (ρ)
Features	−0.22	−0.26	0.82 **
Amenities	−0.20	−0.32	0.19
Incivilities	−0.07	−0.24	−0.16

Abbreviations: PARA = Physical Activity Resource Assessment; MVPA = moderate-to-vigorous physical activity. ** *p* < 0.05.

## Data Availability

The data presented in this study are available on request from the corresponding author (A.N.Z.).

## Supplementary Materials

The following supporting information can be downloaded at: https://www.mdpi.com/article/10.3390/ijerph21050607/s1, Table S1: Crude associations between demographics and physical activity variables in the analytic sample (N = 255); Table S2: Descriptive statistics of PARA measures.
