# Supplementary material for: Exploring the Relationship between Built Environment Attributes and Physical Activity in Lower-Income Aging Adults: Preliminary Insights from a Multi-Level Trial
Source: Int J Environ Res Public Health. 2024 May 9;21(5):607. doi: 10.3390/ijerph21050607 (PMC11121044; doi:10.3390/ijerph21050607)
Supplement: Supplementary file 1 [file ijerph-21-00607-s001.zip › ijerph-2974818-supplementary.pdf]

Table S1: Crude associations between demographics and physical activity variables in the analytic sample (N=255)

		**Median (Q1, Q3)**		
	**n**	**Total walking (min/wk.)**	**Total PA (min/wk.)**	**Total MVPA (min/wk.)**
**Sex**				
Male	67	90 (0, 180)	540 (330, 810)	15 (0, 100)
Female	188	90 (0, 200)	525 (325, 787.50)	5 (0, 97.50)
P (ANOVA)		*0.78*	*0.82*	*0.58*
**Race/ethnicity**				
African American/Black, non-Hispanic	7	70 (0, 150)	385 (360, 680)	0 (0, 50)
Asian, non-Hispanic	40	85 (0, 170)	465 (282.5, 765)	30 (0, 155)
Native American, non-Hispanic	1	225	1435	70
White, non-Hispanic	154	90 (0, 210)	540 (330, 825)	0 (0, 90)
Refused race, non-Hispanic	1	30	650	80
Latino/Hispanic ethnicity	49	90 (0, 155)	495 (315, 765)	30 (0, 90)
Two or more/mixed race	3	120 (0, 280)	1080 (455, 1275)	270 (0, 310)
P (ANOVA)		*0.29*	*0.02***	*0.11*
**Education**				
Less than high school	6	90 (0, 280)	315 (255, 840)	15 (0, 380)
High school or equivalent	43	80 (20. 180)	555 (350, 765)	30 (0, 90)
College	131	90 (0, 210)	495 (290, 825)	0 (0, 90)
Post-graduate	74	82.50 (0, 175)	525 (360, 735)	5 (0, 100)
Other	1	180	765	120
P (ANOVA)		*0.75*	*0.83*	*0.44*
**Participant age group**				
<65 years old	78	90 (0, 180)	540 (360, 765)	30 (0, 120)
Older adults (≥ 65)	177	90 (0, 180)	525 (325, 805)	0 (0, 90)
P (ANOVA)		*0.78*	*0.82*	*0.58*
**Education**				
Less than high school	6	90 (0, 280)	315 (255, 840)	15 (0, 380)
High school or equivalent	43	80 (20. 180)	555 (350, 765)	30 (0, 90)
College	131	90 (0, 210)	495 (290, 825)	0 (0, 90)
Post-graduate	74	82.50 (0, 175)	525 (360, 735)	5 (0, 100)
Other	1	180	765	120
P (ANOVA)		*0.75*	*0.83*	*0.44*
**Housing site**				
Site A	14	50 (0, 180)	495 (270, 825)	25 (0, 150)
Site B	15	125 (30, 210)	810 (360, 900)	0 (0, 60)
Site C	21	150 (0, 240)	450 (300, 630)	0 (0, 60)

Site D	14	170 (0, 315)	547.50 (375, 795)	40 (0, 120)
Site E	16	75 (0, 245)	667.50 (502.5, 832.50)	0 (0, 35)
Site F	15	60 (0, 330)	495 (360, 795)	0 (0, 180)
Site G	35	80 (20, 175)	480 (290, 720)	20 (0, 120)
Site H	32	42.50 (0, 125)	457.50 (195, 750)	50 (0, 100)
Site I	47	75 (20, 120)	545 (360, 845)	10 (0, 120)
Site J	46	100 (20, 160)	480 (285, 960)	37 (0, 120)
P (ANOVA)		*0.31*	*0.44*	*0.91*
**Annual income**				
<$5,000	*2*	60 (0, 120)	812.5 (765, 860)	92.5 (0, 185)
$5,000-9,999	*5*	0 (0, 60)	270 (255, 315)	0 (0, 0)
$10,000-14,999	*17*	150 (0, 210)	765 (510, 965)	50 (0, 210)
$15,000-24,999	*27*	105 (20, 270)	525 (330, 850)	0 (0, 60)
$25,000-34,999	*14*	82.5 (0, 210)	315 (240, 885)	0 (0, 120)
$35,000-49,999	*24*	115 (35, 280)	630 (395, 865)	25 (0, 105)
$50,000-74,999	*29*	10 (0, 135)	450 (345, 615)	0 (0, 45)
>$75,000	*79*	100 (30, 190)	525 (330, 735)	30 (0, 100)
Don't know or refused	*58*	72.5 (20, 140)	490 (325, 735)	42 (0, 130)
P(ANOVA)		*0.11*	*0.66*	*0.26*

Abbreviations: PA = physical activity; MVPA = moderate-to-vigorous physical activity; BMI = body mass index.

Table S2: Descriptive statistics of PARA measures

	Site A	Site B	Site C	Site D	Site E	Site F	Site G	Site H	Site I	Site J
**Total PARs, n**	21	34	32	26	42	14	28	45	18	33
Park, trail, or green space, n (%)	5 (24)	7 (21)	5 (16)	4 (15)	8 (19)	4 (29)	5 (18)	10 (22)	7 (39)	6 (18)
Sports facility or fitness club, n (%)	0 (0)	0 (0)	1 (3)	2 (8)	3 (7)	3 (21)	8 (29)	1 (2)	1 (6)	8 (24)
Community center, n (%)	3 (14)	6 (29)	2 (6)	5 (19)	8 (19)	0 (0)	4 (14)	3 (7)	1 (6)	2 (6)
Church, n (%)	8 (38)	13 (62)	12 (38)	12 (46)	11 (26)	3 (21)	3 (11)	15 (33)	4 (22)	4 (12)
School, n (%)	2 (10)	4 (19)	10 (31)	1 (4)	8 (19)	2 (14)	3 (11)	14 (31)	3 (17)	9 (27)
Plaza, n (%)	2 (10)	0 (0)	1 (3)	1 (4)	2 (5)	0 (0)	1 (4)	0 (0)	0 (0)	0 (0)
Combination[a], n (%)	1 (5)	4 (19)	1 (3)	1 (4)	2 (5)	2 (14)	4 (14)	2 (4)	2 (11)	2 (6)
**Normalized mean scores for PARs audited[b], range 0 to 3**										
Features	0.49	0.45	0.49	0.60	0.45	0.53	0.57	0.53	0.59	0.71
Amenities	1.13	1.26	1.33	1.31	1.21	1.15	1.39	1.16	1.02	1.43
Incivilities	0.30	0.49	0.35	0.32	0.43	0.38	0.32	0.46	0.24	0.33
**Mean counts of PARA elements**										
Features	2.14	2.35	2.31	2.65	2.02	2.43	2.57	2.42	2.67	3.24
Amenities	5.48	6.62	6.19	6.04	5.52	5.36	6.29	5.82	4.67	6.94
Incivilities	2.19	3.35	2.63	2.46	2.62	2.50	2.21	2.89	1.61	2.36
**Averages of PA outcomes (min/week), median (IQR)**										
Walking	50 (0, 180)	125 (30, 210)	150 (0, 240)	170 (0, 315)	75 (0, 245)	60 (0, 330)	80 (20, 175)	42.5 (0, 125)	75 (20, 120)	100 (20, 160)
Total PA	495 (270, 825)	810 (360, 900)	450 (300, 630)	547.5 (375, 795)	667.5 (502.5, 832.5)	495 (360, 795)	480 (290, 720)	457.5 (195, 750)	545 (360, 845)	480 (285, 960)
MVPA	25 (0, 150)	0 (0, 60)	0 (0, 60)	40 (0, 120)	0 (0, 35)	0 (0, 180)	20 (0, 120)	50 (0, 100)	10 (0, 120)	37 (0, 120)

Abbreviations: PARA = Physical Activity Resource Assessment; PARs = Physical activity resources; PA = Physical activity; MVPA = Moderate-to-vigorous physical activity

[a] "Combination" is used to describe a PAR that has a combination of two or more of the listed resources.

[b] Normalized mean scores were calculated by first calculating the mean of the items in each domain for each PAR and averaging across all of the PARs in a site to get an overall mean for each PARA domain. This results in scores on a scale of 0 to 3 for each domain.
